# Simplification of Caribbean Reef-Fish Assemblages over Decades of Coral Reef Degradation

**DOI:** 10.1371/journal.pone.0126004

**Published:** 2015-04-14

**Authors:** Lorenzo Alvarez-Filip, Michelle J. Paddack, Ben Collen, D. Ross Robertson, Isabelle M. Côté

**Affiliations:** 1 Unidad Académica de Sistemas Arrecifales, Instituto de Ciencias del Mar y Limnología, Universidad Nacional Autónoma de México, Puerto Morelos, Quintana Roo, México; 2 Department of Biological Sciences, Simon Fraser University, Burnaby, BC, V5A 1S6, Canada; 3 Department of Biology, Santa Barbara City College, Santa Barbara, California, 93109, United States of America; 4 Centre for Biodiversity and Environment Research, University College London, Gower Street, London, WC1E 6BT, United Kingdom; 5 Smithsonian Tropical Research Institute, Balboa, Republic of Panamá; The Australian National University, AUSTRALIA

## Abstract

Caribbean coral reefs are becoming structurally simpler, largely due to human impacts. The consequences of this trend for reef-associated communities are currently unclear, but expected to be profound. Here, we assess whether changes in fish assemblages have been non-random over several decades of declining reef structure. More specifically, we predicted that species that depend exclusively on coral reef habitat (i.e., habitat specialists) should be at a disadvantage compared to those that use a broader array of habitats (i.e., habitat generalists). Analysing 3727 abundance trends of 161 Caribbean reef-fishes, surveyed between 1980 and 2006, we found that the trends of habitat-generalists and habitat-specialists differed markedly. The abundance of specialists started to decline in the mid-1980s, reaching a low of ~60% of the 1980 baseline by the mid-1990s. Both the average and the variation in abundance of specialists have increased since the early 2000s, although the average is still well below the baseline level of 1980. This modest recovery occurred despite no clear evidence of a regional recovery in coral reef habitat quality in the Caribbean during the 2000s. In contrast, the abundance of generalist fishes remained relatively stable over the same three decades. Few specialist species are fished, thus their population declines are most likely linked to habitat degradation. These results mirror the observed trends of replacement of specialists by generalists, observed in terrestrial taxa across the globe. A significant challenge that arises from our findings is now to investigate if, and how, such community-level changes in fish populations affect ecosystem function.

## Introduction

Habitat degradation modifies the structure of ecological assemblages by altering biotic interactions and ecosystem dynamics. The extent of a species’ reliance on specific habitat features is one of the characteristics that should determine how well it will fare under most scenarios of habitat change [1,2]. While habitat specialists should be relatively sensitive to the degradation of their preferred habitat, generalists may be less affected, or could even benefit from new habitat arrangements or from the reduction in abundance or disappearance of other species (i.e., predators and competitors) via ecological compensation [3,4]. Across various taxa and geographical regions, specialist species appear to be declining and experiencing higher extinction risk in response to habitat change compared to generalist species, leading to homogenization and simplification of communities over time [5]. Measuring how species or groups of functionally similar species within communities differ in their responses to habitat change is therefore crucial to understand and predict the consequences of habitat loss and environmental degradation for species assemblages and ecosystem functioning. In fact, trends in the abundance of specialist species are now used as national and international indicators of development sustainability (e.g., [6]).

Coral reefs are changing rapidly, worldwide. This is particularly evident in the Caribbean where the structural complexity of many reefs has greatly declined, due to loss of coral cover and changes in the composition of coral assemblages [7–9]. The ecological repercussions of long-term decline in architectural complexity are likely to be substantial. For many reef fishes and invertebrates, the risk of predation is influenced by access to refuges; thus, the richness, abundance and biomass of these species are influenced by habitat complexity [10,11]. The consequences for coral reef fishes are of particular concern because this group plays key roles in ecosystem functioning (e.g., [12,13]) and reef fisheries are important for the livelihood of many human coastal communities [14]. In a large-scale meta-analysis, Paddack et al. [15] found that since the late 1990s the overall density of Caribbean reef fishes has declined consistently across the region. This reduction was attributed in part to declines in coral cover and reef complexity, which began as early as the 1970s [7,16].

Species seldom all respond similarly to changes in habitat. In the Indo-Pacific region, for example, a variety of species in a range of taxa depend nearly exclusively on live coral for food or shelter and, predictably, these specialists have often declined more precipitously in response to coral loss than less dependent species [10,17–19]. In the Caribbean, very few reef fishes are as heavily reliant on live corals as their Indo-Pacific counterparts [20]. However, this does not mean that all Caribbean reef fishes are generalists. Ecological specialization is not a dichotomous state but instead ranges along a continuum [21]. Caribbean reef fishes vary in terms of the extent to which they use non-reef as well as reef habitats, and the extent to which they clearly associate with specific features of reef structure or microhabitats (e.g., [22–24]). The recent changes in Caribbean reef fish density could therefore have been accompanied by shifts in relative abundance of slightly more and slightly less specialised species that are more subtle than those observed in the Indo-Pacific region.

The rate of change in population size is one of the most sensitive metrics for assessment of long-term biodiversity change [25–28]. Changes in abundance provide information about both variability and quantity of biodiversity (25,27), and can be used to detect shifts in community composition (26) and to infer changes in habitats and/or intensity of threats, such as exploitation (25). Here, we test the hypothesis that fishes that are more specialised in terms of coral reef habitat use have declined more in abundance than have habitat-generalists at the same locations across reef distributed throughout much of the Caribbean. We apply a novel method designed to measure the state of biodiversity based on species population trends [29] to a large, regional-level database of temporal variation in the abundances of Caribbean reef fishes to examine large-scale trends of change in habitat-specialists and habitat-generalists. Furthermore, by examining the trends for fished and unfished species in those two groups, we test whether any differences in trajectories are attributable to fishing rather than causes such as changes in habitat structure.

## Methods

### Database and species grouping

We used a subset of the database that was compiled by Paddack et al. [15] of temporally replicated, quantitative data on Caribbean reef fish density (individuals m^-2^) from *in situ* surveys conducted by trained scientific observers (see details in Paddack et al. [15]). To be included, each study needed to have (i) reported a density estimate of at least one reef fish species from a reef site within the Caribbean region, (ii) surveyed the same species at the same site over more than one year, and (iii) replicated measurements during each survey. In the current analysis we used only common species, which we defined as those observed in at least 50% of the surveys in a time series. With this restriction, we aimed to control for the potentially influential effect of zero-density values in short (2–5 years) time series.

Specialization can be quantified by measuring the narrowness of use of a particular gradient of resource or habitat [25]. Because no Caribbean fish species relies exclusively on live corals and very few are closely associated with live coral [30,31], we based our specialization categories on the strength of association with coral reef habitat in general. Thus we classed as ‘specialists’ those species that are only found on coral reefs, and as ‘generalists’, those species that are associated with a broader range of habitat types, including less complex habitats such as seagrass beds, gorgonian fields, sponge beds and macroalgal stands. For our analyses we used the specialist/generalist assignation of species made by Luiz et al. [32] who categorized fish species using data on habitat-use and latitudinal-range obtained from bibliographic sources and online databases, supplemented with field records (see Luiz et al. [32] for details). Species were classified as specialists or generalists prior to analyses and before exploring the general trends from each group in the database. In total we categorized 81 species from 27 families as habitat-specialists and 80 species from 26 families as habitat-generalists (S1 Table). Fish species in each of the two habitat-use groups were also separated into two sub-groups based on their level of exploitation. We obtained the fishing status categorization from Paddack et al. [15] and FishBase [33]. Unfished species included those that are not marketed, have unknown fishing status, or are targeted only lightly by the aquarium trade. Fished species included those that are marketed as food-fish or are heavily targeted by the aquarium trade.

The majority of the data came from studies that methodically collected abundance estimates for many species in each reef fish assemblage (see [15]). As such, our study is unlikely to over-represent species of special interest (e.g., threatened species), which is a common caveat in studies of this kind (e.g., [29, 34]).

### Trend analysis

To generate overall density trends for habitat-specialist and habitat-generalist species we used an aggregated index of change in abundance. This index of abundance was developed to provide scientists, policy-makers, and the general public with information on trends in the abundance of vertebrate populations across the globe [35]. This index represents an effective heuristic instrument for indicating trends in global biodiversity [29], and has been adopted as a key indicator of the state of global biological diversity at the international level [28,36]. In addition, it can be used to evaluate broad scale trends for biogeographic realms, biomes, habitats, and particular taxonomic groups (e.g., [29, 34]).

We outline the main steps for abundance-index computation here, although a more thorough description of the method and equations is provided in S1 Appendix. For each time series, the change in density from each year to the next was calculated. In the case of incomplete time series, zero values were replaced by one percent of the mean population value for the whole time series, and missing values for yearly censuses were derived from interpolation of the preceding and the subsequent years with measured values (see [29]). Missing value interpolations were applied to most of the time series, given the gaps in data collection in the majority of monitoring schemes (S1 Fig). Two different methods were then used to generate estimates of change for each time series: log-linear models (the chain method in Loh et al. [35]) were used to compute values for short time series (n < 6 time intervals; 70% of the total), and generalized additive models were used to compute values for all other time series. The use of this smoothing approach accounts better for non-linear variation [29,37].

Trends of all populations of the same species were averaged to produce species-specific trends. The average rate of change in each year across all species was then calculated. Finally, the average annual species change in each year was chained to the previous year to generate a continuous index, starting with an initial value of 1 in the first year of the database, which was 1980 in this study. All populations within species were given equal weight in the calculation of species-specific trends because we had no information on the relative importance of different populations to region-wide species abundance. Similarly, each species contributed equally to the calculation of the index. An advantage of giving equal weighting to all species is that common species or highly abundant populations do not have a disproportionate influence on the index trajectory. Separate indices were computed for habitat-specialist and habitat-generalist species and then, for fished and unfished species within each of those two habitat-use groups.

The uncertainty of the index estimates was assessed with bootstrapped 95% confidence intervals (CI). For each year, 1000 index values were calculated from randomly sampled species-specific population changes [35]. We considered a divergence of the overall trend as significant when the CI did not encompass the overall population baseline (i.e., Index of Abundance = 1).

## Results and Discussion

**Fig 1:**
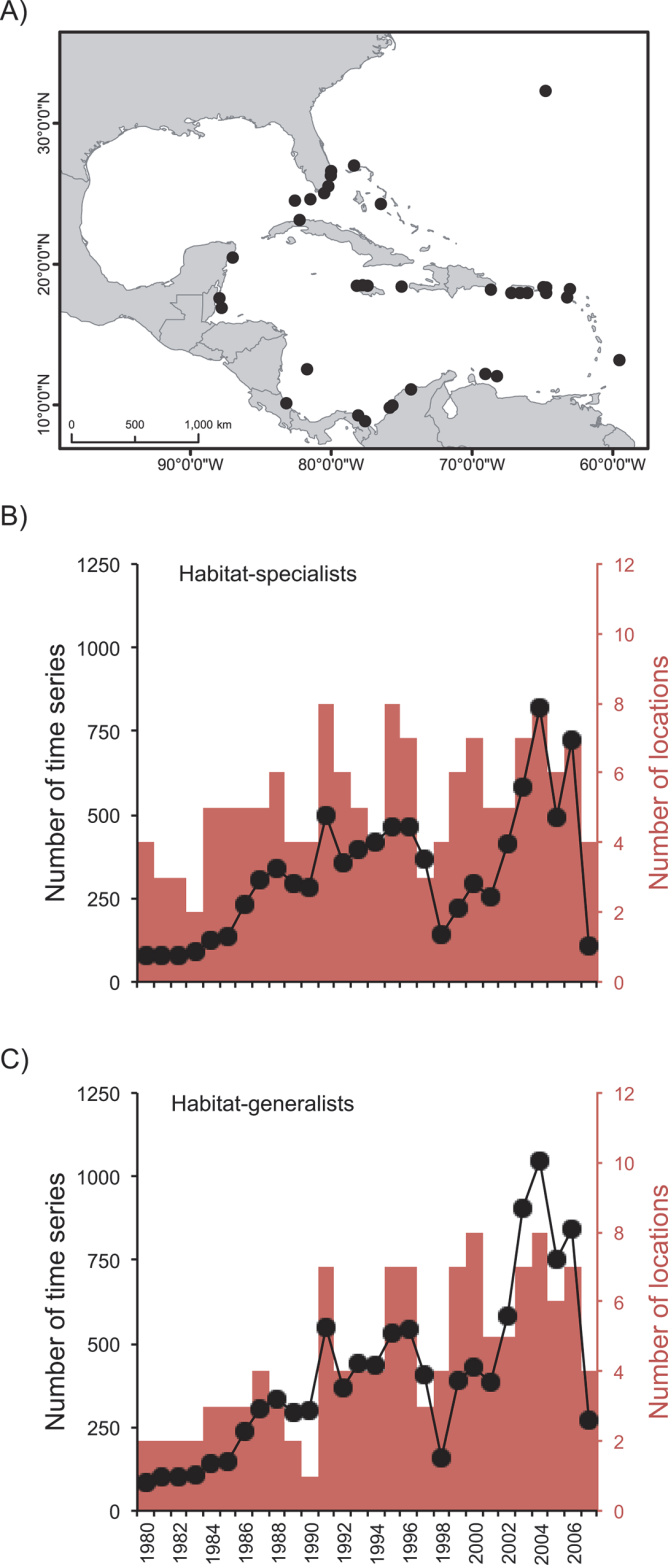
Spatial and temporal distribution of time-series of abundance of Caribbean reef fishes. (A) Location of the study sites is shown as black dots (note that one dot usually represents multiple reefs). Numbers of time series (black dots) and sites (red bars) included in the Index of Abundance for (B) habitat-specialists, and (C) habitat-generalists.

**Fig 2:**
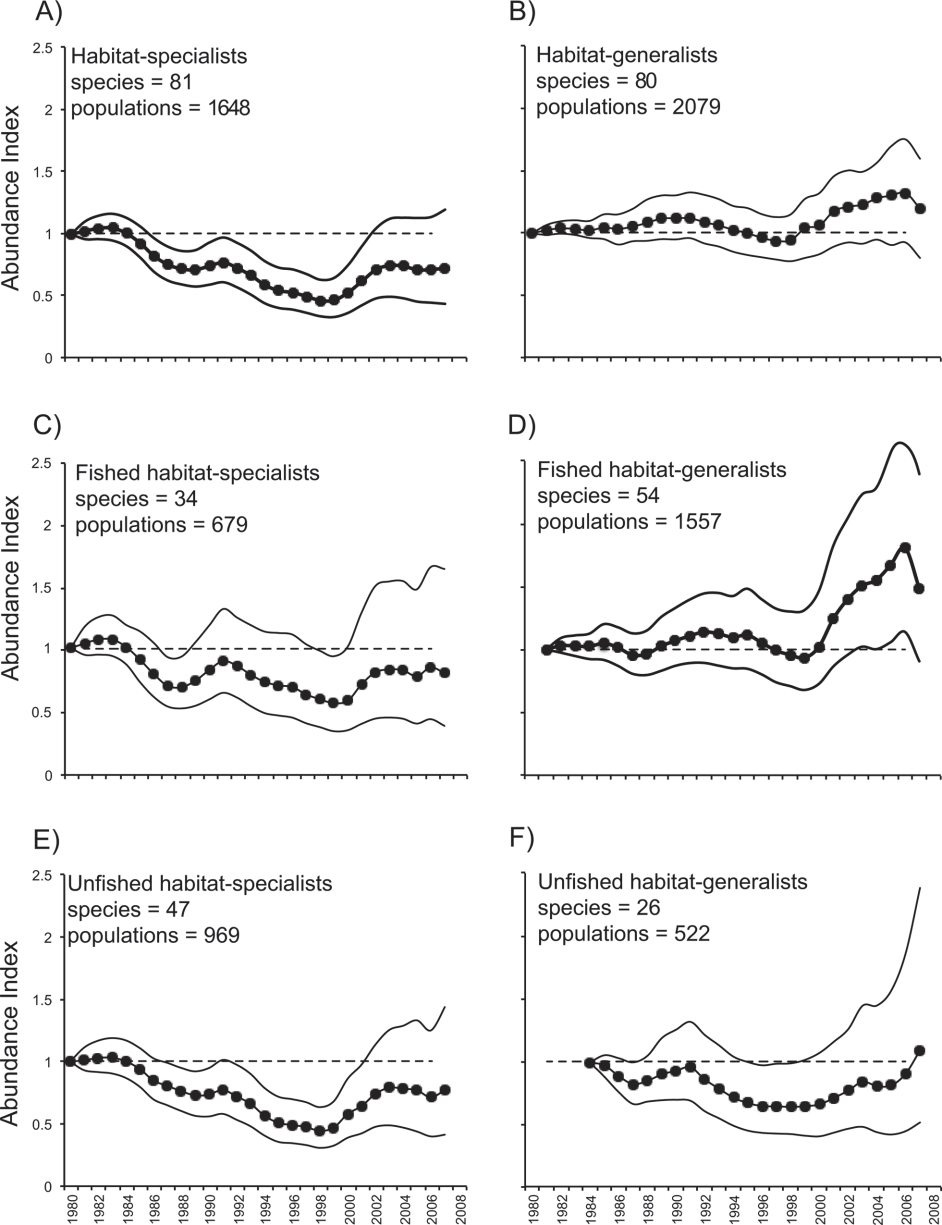
Temporal trends in overall Caribbean reef fish abundance, relative to 1980 as depicted by the Index of Abundance. Plots show average change (+/- 95% CI, see Methods), with dashed line at y = 1 indicating the 1980 value. The numbers of species and populations used to generate each panel are indicated. (A) all habitat-specialists; (B) all habitat-generalists; (C) habitat-specialists targeted by fisheries; (D) habitat-generalists targeted by fisheries; (E) unfished habitat-specialists; and (F) unfished habitat-generalists.

Our analysis included 3727 population trends for 161 common reef fishes from sites distributed throughout the wider Caribbean region (Fig 1A). As is common in this type of study, the number of sites and times series represented in the analyses tended to increase over time (Fig 1B and 1C); however, the geographical representativeness of the data has been regionally comprehensive since the 1990s (S1 Fig). We found that abundance trends differed markedly between habitat-generalists and habitat-specialists. The abundance of specialists began declining in the mid-1980s, reaching a low of ~60% of the 1980 baseline abundance by the late-1990s (Fig 2A). Average abundance and variation have increased somewhat since the early 2000s, although the average is still well below the baseline level of 1980. In contrast, the abundance of generalist species has remained relatively stable since 1980, with a marked but non-significant upswing since 2000 (Fig 2B). The trends of change of specialists and generalists were significantly different from each other between late-1980s and late-1990s, but the differences became less apparent during the 2000s due to the high variability associated with both trends (see CIs in Fig 2A and 2B; and S2 Fig). The patterns uncovered here are not due to the presence of different time series in different parts of the study period. Monitoring at many sites used in the analyses ceased or began in the late-1990s (S1 Fig); however, when only time series that span most of the study duration are used, the average trends are similar, albeit with larger confidence intervals owing to the smaller sample sizes (S3 Fig).

The decline of habitat-specialist fishes we find here began soon after periods of rapid declines in coral cover and reef complexity during the early 1980s [7,16,38] (S4 Fig). The widespread mortality of morphologically complex, branching *Acropora* corals in the Caribbean, owing to white-band disease starting in the mid-1970s [39], likely accounts for some of these early changes to reef habitats. Caribbean *Acropora* reefs tend to support higher fish abundance and species richness than areas with lower structural complexity [40]. Thus, it is likely that the near-disappearance of these corals from many areas reduced drastically the amount of a major structural habitat feature throughout the 1980s [39], and affected populations of a number of reef fishes. While it is not possible to determine whether the decline of specialist species began before 1980, the ~4–5 years of relative stability at the start of the temporal trend suggest that populations of those species were not already undergoing a steep decline at that time (Fig 2A). Thus, specialist species may have experienced a lagged response to the loss of reef complexity, which started a few years earlier ([7,16]; Fig 2A). Several factors may have contributed to this delayed effect: (i) a lag in the degradation of microhabitat structure sufficient to have a measureable effect on specialist populations; (ii) reduced recruitment by fishes that need fine-scale structure as settlement microhabitat; and (iii) reductions in survivorship and/or reproductive output of specialists, owing to loss of shelter or food resources [10,17,41].

**Fig 3:**
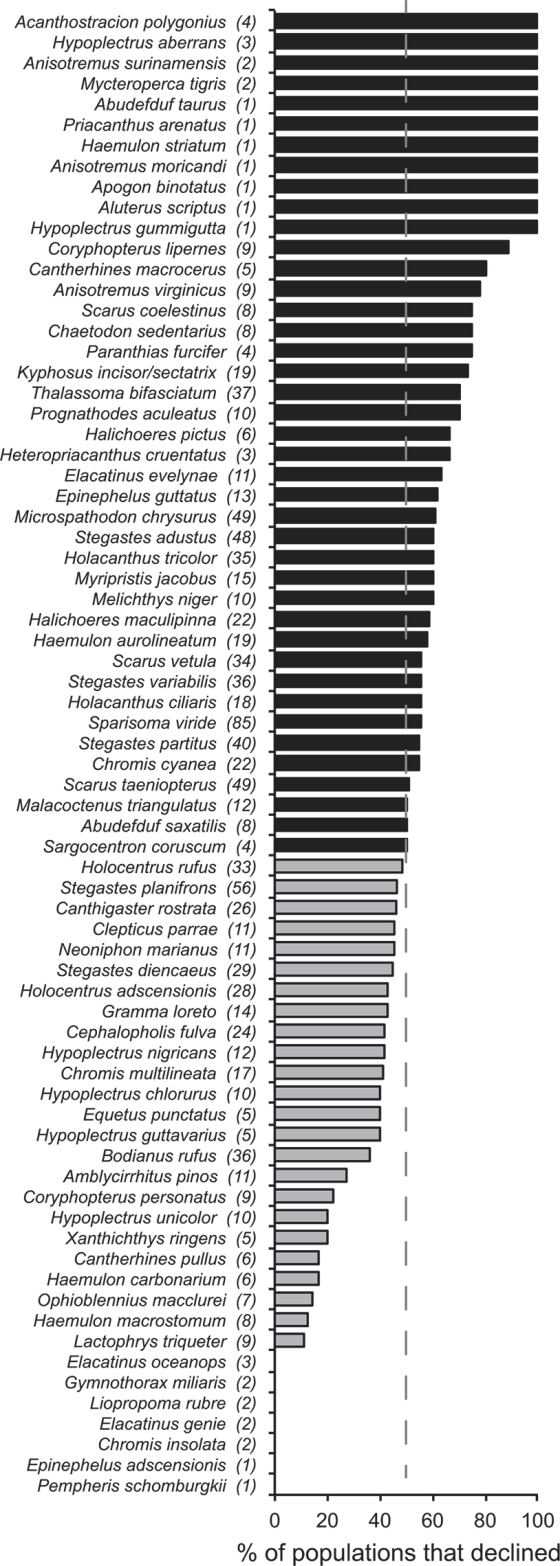
Percentage of time-series (= populations) of each habitat-specialist species that declined between the first and the last year of their monitoring period between 2000 and 2007. This time period spans the apparent partial recovery of specialist species in the early 2000s (see Fig 2A). The total number of time-series by habitat-specialist species is indicated in parentheses. The 72 species are ranked from the highest to the lowest percentage of declining time-series. Habitat-specialist species with 50% or more of declining time-series (black bars), and less than 50% (grey bars) are identified. In total, 557 populations declined, 446 increased and 64 were stable (no change). Note that this figure does not show the magnitude of the changes.

**Fig 4:**
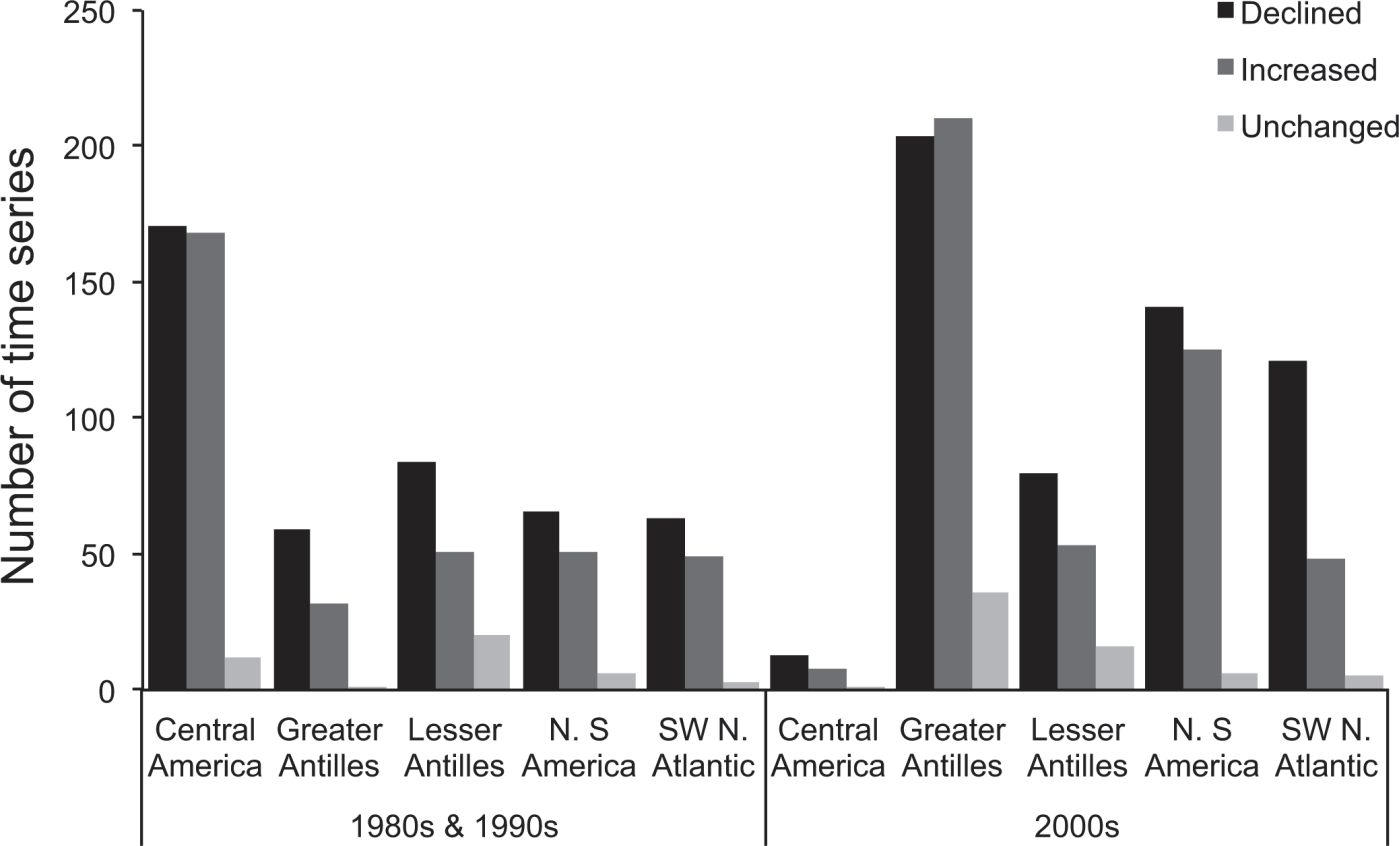
Number of time series (= populations) of habitat-specialist species that show a decline, a recovery or remained stable between the first and last year of their monitoring period. The data are presented by geographical region (as in S1 Fig) and for two time periods (1980s-1990s, and 2000s). These two time periods were selected based on the overall trend of Fig 2A, which shows partial recovery of specialist species in the early 2000s. In the 1980s-1990s, a total of 443 populations declined, 351 increased and 42 were stable (no change). In the 2000s, a total of 562 species declined, 441 increased, and 64 remained stable. Note that this figure does not show the magnitude of the changes.

The decline in abundance of habitat-specialists continued steadily through the 1990s, with some evidence of modest recovery since 2000 (Fig 1A). This pattern of increase is unlikely to be the result of any improvement in habitat quality on Caribbean reefs, as it occurred in a decade when, at the regional scale, coral cover remained relatively low and reef architectural complexity declined steeply [7,16,38] (S4 Fig). The overall increase in the index values of specialist species in the 2000s is accompanied by an increasing variance (Fig 2A): while some populations were recovering, many more were continuing to decline (Figs 3 and 4). Biological traits and geographic distribution are usually poor predictors of population change on a broad scale (e.g., [42,43]); hence we did not attempt to investigate the identity or location of the species driving changes in the abundance of either specialists or generalists. However, we found no evidence that the increasing trend during the 2000s is related to positive changes in the abundance of a discrete subset of specialist species: most species show a mixture of declining and increasing populations during the 2000s (Fig 3). There were some geographic differences in recovery, with the Greater Antilles posting relatively more recovering than declining populations of habitat-specialists during the 2000s (Fig 4). This pattern contributed to the large confidence intervals that characterize all of the overall trends (Fig 2) in the last decade of the study period. Understanding the reasons for this geographic pattern will require further investigation.

In contrast to specialist fishes, the abundance of generalists on Caribbean reefs has remained relatively constant throughout the three decades spanned by this study (Fig 2B). This pattern suggests that fishes in this group were not negatively affected by changes occurring on coral reefs, and some may even have benefited from the reduced abundance of some other species (e.g., of food competitors) and/or from the loss of reef architectural complexity [3, 5, 44]. Generalist reef fishes may have relatively plastic preferences for settlement microhabitats, allowing them to recruit more successfully onto degraded reefs and alternative habitats than habitat specialists. For example, parrotfishes that sometimes exhibit strong recruitment associations with specific coral microhabitats (e.g., [24]) also recruit onto macroalgae on reefs with low coral cover [45]. It has been suggested that fishes at higher trophic levels (which are mostly generalists; S1 Table) might experience lagged reductions due to declines in habitat-dependent prey fishes [10]. However, we did not find any evidence in support of this in our regional analysis. The abundance of generalist species remained relatively constant independently of the declines in the abundance of specialist species.

Fishing could be inferred to drive overall changes in Caribbean reef fish assemblages if fished species declined while unfished species did not. We found no evidence for such a fishing effect. Fished and unfished specialists show much the same abundance dynamics (Fig 2C vs. 2E). Note, however, that the uncertainty is high for exploited specialist fishes, probably due to the relatively low sample size. This observation supports our view that declines in the abundance of specialist species through the 1980s and 1990s were likely a consequence of reef degradation. The trends of fished and unfished generalists are not significantly different from each other (CIs overlap in Fig 2D vs. 2F). Although the uncertainty in the trends of both fishes and unfished habitat-generalists is large, particularly over the last five years of the series (Fig 1F), fished generalists increased distinctly more than unfished generalists during the last third of the study period (Fig 2D). In a previous study, Paddack et al. [15] also found similar rates of change of fished and unfished species in general, supporting the notion that fishing has not been a main driver of the recent changes in the abundance of reef fishes. It remains possible that fishing pressure and indiscriminate fishing methods may have affected unfished species indirectly through bycatch or by altering food-web dynamics (e.g., predation release; [46]). However, such effects would be difficult to assess with our data.

The slight increase in abundance of many fished generalist species in the last decade of the study is surprising because it is produced largely by significant increases in species of commercial importance such as groupers and snappers (S5 Fig). Changes in fisheries management are unlikely to be responsible for this pattern. The number and extent of species, gear and other effort restrictions vary widely across Caribbean nations [47]. For this reason, and because of poor compliance and enforcement remain problems in the region [48], it seems unlikely that a regional-scale increase in abundance of exploited generalist species is the result of multiple independent changes in national catch and effort regulations. Similarly, it is unlikely that the increasing use of marine protected areas (MPAs) as fishery management tools (cf., [49]) explains the increase in abundance of fished generalists. Only 8% of our time series stemmed from monitoring sites in marine protected areas, and the number of populations from such sites actually decreased over time (S6 Fig). The relative increase in abundance of fished species in the last third of our study period may be a reflection of the time horizon of our study. Many of the populations of exploited generalist species likely were already heavily depleted well before the start of our study [50,51]. Hence, the increase we recorded since the turn of the millennium is likely to be relatively small in comparison to original population sizes. That is, a ‘shifting baseline’ effect, due to a heavily depressed level at the start of the study (1980), may have amplified the *apparent* size of any subsequent increase. Although it remains difficult to understand why this mechanism would affect fished generalists more than their unfished counterparts, it is important to remember that the apparent increase in abundance of fished generalists does not necessarily indicate healthy populations of commercially important species, or that the negative effects of fishing have ceased in the Caribbean. Fishing often truncates the age and size structure of populations [52]. Thus, the trend of commercially important generalists might be driven by increases in the abundance of small-sized individuals (e.g., juveniles) rather than increases in individual size and biomass that are needed to effect population recovery. Shifts in species composition from large-bodied to smaller-bodied species via a compensatory response are also possible.

Our results indicate that Caribbean reef-fish assemblages have been experiencing profound changes in community composition since 1980, probably largely due to habitat degradation. We found evidence of an apparent replacement of habitat-specialists by generalist species over a 30-year period. Such a shift is symptomatic of disturbed ecosystems around the world [4,6] and, in some cases, results in large-scale spatial homogenisation of previously diverse species assemblages [5, 53]. The ecological replacement of specialized species by generalists could have consequences for ecosystem integrity and function. For example, the loss of specialist species is likely to reduce the variability in community responses to disturbances and environmental change, and modify species interaction networks, which could result in the loss of key ecosystem functions [1,5,21,54]. Further research assessing the links between habitat specialisation and ecosystem functioning, in terms of species replacement triggering trophic cascades, altering energy flux, and changing functional redundancy, is crucial to fully understand the consequences of habitat degradation for coral reef diversity and for the resources they provide to humanity.

## Supporting Information

S1 Appendix Calculation of the Abundance Index. (PDF)

S1 Fig Time series of data available from different census sites that, together, provided the 3727 populations included in this study. Each line showed the time span of a time series. Time series are colour-coded by sub-region of the Caribbean to show the geographic spread of the data.
(PDF)

S2 Fig Significance (*p* values) of the annual difference between the trends of habitat-generalists and habitat-specialists shown in Fig 2A and 2B. *p* values were derived from T tests comparing the final index values and 95% CIs of the specialist and generalist trends. The dashed line shows the critical value below which differences are statistically significant.
(PDF)

S3 Fig Temporal trends in the Abundance Index (+/- 95% CI, see Methods for calculations) of habitat-specialist (left column) and habitat-generalist (right column) Caribbean reef-fish species in long time-series that either stop in 1998 (top row) or span 1998 (middle row). The baseline year is indicated by a dashed line. Panels (A) and (B) from Fig 2 are shown to facilitate the visual interpretation.
(PDF)

S4 Fig Long-term trajectories of change in coral cover, reef rugosity and habitat-specialist fishes on Caribbean reefs. A) Region-wide changes in mean coral cover and reef rugosity based on a meta-analysis of ecological studies across the Caribbean from 1977 to 2008 (Redrawn from Alvarez-Filip et al. 2011; *Global Change Biol*. 17:2470–2477). B) Temporal trends in the overall abundance of habitat-specialist fishes, relative to 1980 as depicted by the Abundance Index (Redrawn from Fig 2A). The grey dotted line indicates the year 2000, when at the regional scale, the abundance of habitat-specialist fishes started to recover but coral cover and reef rugosity continued to decline. The two panels derive from different data sources due to the lack of site overlap between the habitat (coral cover and reef rugosity) and fish datasets.
(PDF)

S5 Fig Temporal trends in aggregate abundance (Abundance Index, with 95% CI) of two major taxa of commercially important Caribbean reef fishes: Serranidae (groupers) and Lutjanidae (snappers). Baseline year is 1986 (dashed line at y = 1).
(PDF)

S6 Fig Number of populations (i.e., time series) collected from inside (red bars) and outside (blue bars) Marine Protected Areas in the Caribbean in each year of the study. Between 1980 and 1988 all times series for inside MPAs are from only one study in Florida, USA. From 1991 to 2007, the number of studies contributing information for sites inside MPAs ranged between one and two, and represented only three other countries/territories (Saba, Costa Rica and Curaçao). Due to the scatter spatial and temporal distribution of the data, it was not possible to further explore the trends of change inside MPAs with the Abundance Index.
(PDF)

S1 Table Species included in this study, their habitat categorisation according to the use of reef habitats. Specialists use only coral reef habitats, and generalists use coral reefs as well as one or more other coastal habitats (classification from Luiz et al. 2012). Fishing status is from Paddack et al. (2009). See Methods for description.
(PDF)
