# Supplementary material for: Simplification of Caribbean Reef-Fish Assemblages over Decades of Coral Reef Degradation
Source: PLoS One. 2015 Apr 14;10(4):e0126004. doi: 10.1371/journal.pone.0126004 (PMC4397080; doi:10.1371/journal.pone.0126004)
Supplement: S1 Appendix — (PDF) [file pone.0126004.s001.pdf]

Online supplementary material

Calculation of the Abundance Index

We used 2 different methods to generate index values: a chain method (Loh et al. 2005) and a generalized additive modeling technique (Fewster et al. 2000; Buckland et al. 2005). We followed Loh et al. (2005) to implement the chain method; however, as per Collen et al (2009) we calculated the logarithm of the ratio of population abundance for successive years (*d*), rather than for 5 yearly intervals:

$$d_t = \log_{10}\left(N_t / N_{t-1}\right), \tag{1}$$

where *N* is the population measure and *t* is the year. One percent of the mean population abundance value for the whole time series was added to all years when *N* = zero. Missing values were imputed with log-linear interpolation (no values were extrapolated):

$$N_i = N_p\left(N_s / N_p\right)^{[(i-p)/(s-p)]}, \tag{2}$$

where *i* is the year for which the value is interpolated, *p* is the preceding year with a measured value, and *s* is the subsequent year with a measured value. For species with more than one time series, the mean value of d_t was calculated across all time series for that species. Species-specific values for d_t were combined:

$$\bar{d}_t = \frac{1}{n_t}\sum_{i=1}^{n_t} d_{it}\,. \tag{3}$$

The index value (*I*) was then calculated in year *t* as

$$I_t = I_{t-1}10^{\bar{d}_t} \tag{4}$$

with the index value set to 1 in 1980, the first year of the time series. Insufficient data were available to run the index prior to 1980 or continue it beyond 2007 (because of a lag in publication of data).

Time series with $n<6$ were analyzed with the chain method. Following Collen et al. (2009) for all other time series, we implemented a generalized additive model (GAM), specified with the mgcv package framework in R (Wood 2006). For each time series we:

1. fitted a GAM on observed values with $\log_{10}(N_t)$ as the dependent variable and year (*t*) as the independent,

2. set the smoothing parameter to the length of the population time series divided by 2 (Wood 2006),
3. selected the smoothing-parameter value by comparing the estimated degrees of freedom when the smoothing parameter was successively incremented by 1,
4. used fitted GAM values to calculate predicted values for all years (including those with no real count data), and
5. averaged and aggregated *d* values from the imputed counts as described above.

A GAM framework might be advantageous in long-term trend analysis because it allows change in mean abundance to follow any smooth curve, not just a linear form (Fewster et al. 2000). The GAM method has greater flexibility for drawing out the long-term nonlinear trends that are generally not elicited in the discrete annual estimates of the chain method. We weighted the analysis, as described above, with species with more than one time series averaged across all the time series for that species. We combined specific values with geometric means at each time point and calculated the index.

Calculation of confidence limits

We used a bootstrap resampling technique to generate confidence limits around index values. To calculate a bootstrap replicate, for each interval, $t-1$ to t, a sample of n_t species-specific values of d_t was selected at random with replacement from the n_t observed values. We implemented the bootstrap procedure 10,000 times and used the bounds of the central 9,500 I values for each year to represent the 95% confidence interval for the index in that year (Loh et al. 2005). Setting the base year (1980) to unity did not mean there was no uncertainty associated with it, rather that the uncertainty was inherited by the rest of the values in the series.

Literature Cited

Buckland, S. T., A. E. Magurran, R. E. Green, and R. M. Fewster. 2005. Monitoring change in biodiversity through composite indices. Philosophical Transactions of the Royal Society of London B **360**:243-254.

Crawley, M. J. 2002. Statistical computing: an introduction to data analysis using S-Plus. Wiley, Chichester, United Kingdom.

Collen B, Loh J, Whitmee S, McRae L, Amin R, Baillie JEM (2009). Monitoring change in vertebrate abundance: the Living Planet Index. Conserv Biol 23: 317-27.

Fewster, R. M., S. T. Buckland, G. M. Siriwardena, S. R. Baillie, and J. D. Wilson. 2000. Analysis of population trends for farmland birds using generalized additive models. Ecology **81**:1970-1984.

Loh, J., R. E. Green, T. Ricketts, J. F. Lamoreux, M. Jenkins, V. Kapos, and J. Randers. 2005. The living planet index: using species population time series to track trends in biodiversity. Philosophical Transactions of the Royal Society of London B **360**:289-295.

R Development Core Team. 2006. R: language and environment for statistical computing. R Foundation for Statistical Computing, Vienna.

Wood, S. N. 2006. Generalized additive models: an introduction with R. Chapman & Hall/CRC, Boca Raton, Florida.
