# Supplementary material for: Simplification of Caribbean Reef-Fish Assemblages over Decades of Coral Reef Degradation
Source: PLoS One. 2015 Apr 14;10(4):e0126004. doi: 10.1371/journal.pone.0126004 (PMC4397080; doi:10.1371/journal.pone.0126004)
Supplement: S1 Fig — Each line showed the time span of a time series. Time series are colour-coded by sub-region of the Caribbean to show the geographic spread of the data. (PDF) [file pone.0126004.s002.pdf]

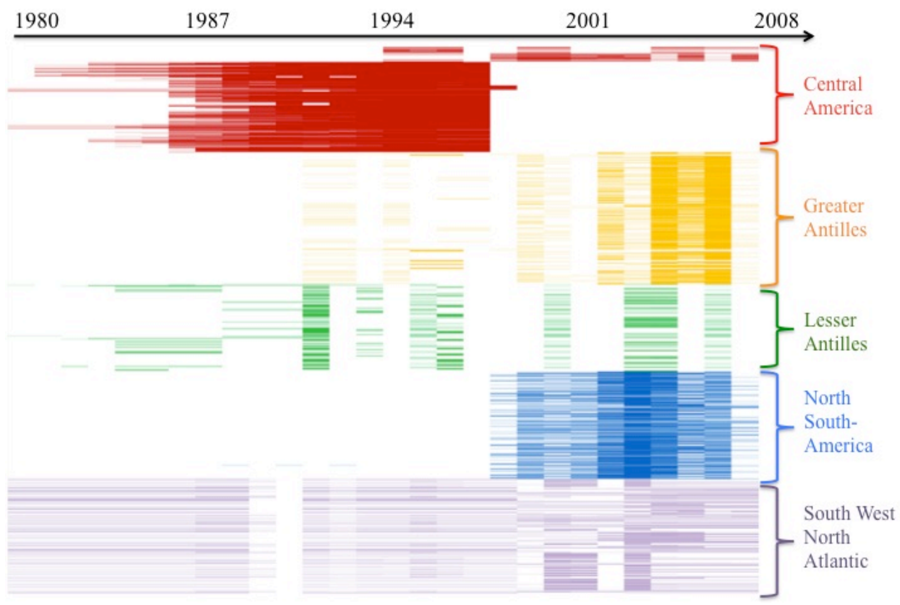

Figure S1. Time series of data available from different census sites that, together, provided the 3727 populations included in this study. Each line showed the time span of a time series. Time series are colour-coded by sub-region of the Caribbean to show the geographic spread of the data.
