# Supplementary material for: Simplification of Caribbean Reef-Fish Assemblages over Decades of Coral Reef Degradation
Source: PLoS One. 2015 Apr 14;10(4):e0126004. doi: 10.1371/journal.pone.0126004 (PMC4397080; doi:10.1371/journal.pone.0126004)
Supplement: S2 Fig — p values were derived from T tests comparing the final index values and 95% CIs of the specialist and generalist trends. The dashed line shows the critical value below which differences are statistically significant. (PDF) [file pone.0126004.s003.pdf]

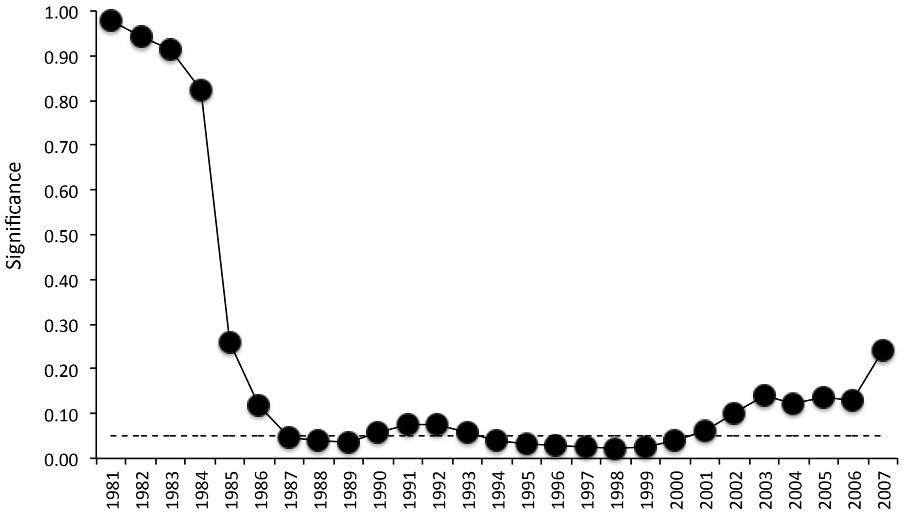

Figure S2. Significance (*p* values) of the annual difference between the trends of habitat-generalists and habitat-specialists shown in Fig 2A and B. *p* values were derived from T tests comparing the final index values and 95% CIs of the specialist and generalist trends. The dashed line shows the critical value below which differences are statistically significant.
