# Supplementary material for: Simplification of Caribbean Reef-Fish Assemblages over Decades of Coral Reef Degradation
Source: PLoS One. 2015 Apr 14;10(4):e0126004. doi: 10.1371/journal.pone.0126004 (PMC4397080; doi:10.1371/journal.pone.0126004)
Supplement: S3 Fig — The baseline year is indicated by a dashed line. Panels (A) and (B) from Fig 2 are shown to facilitate the visual interpretation. (PDF) [file pone.0126004.s004.pdf]

As can be seen in Figure S1, monitoring at many sites either ceased or began around 1998. We explored the effect of this change-over of sites by analysing only the time series that span this time period. The patterns are shown in Figure S3 and are consistent with the trend shown by time series ending in 1998 and by the complete data set (Figs 2A & B in main paper, reproduced below), indicating that the turnover of monitoring sites did not drive the main findings of our analyses.

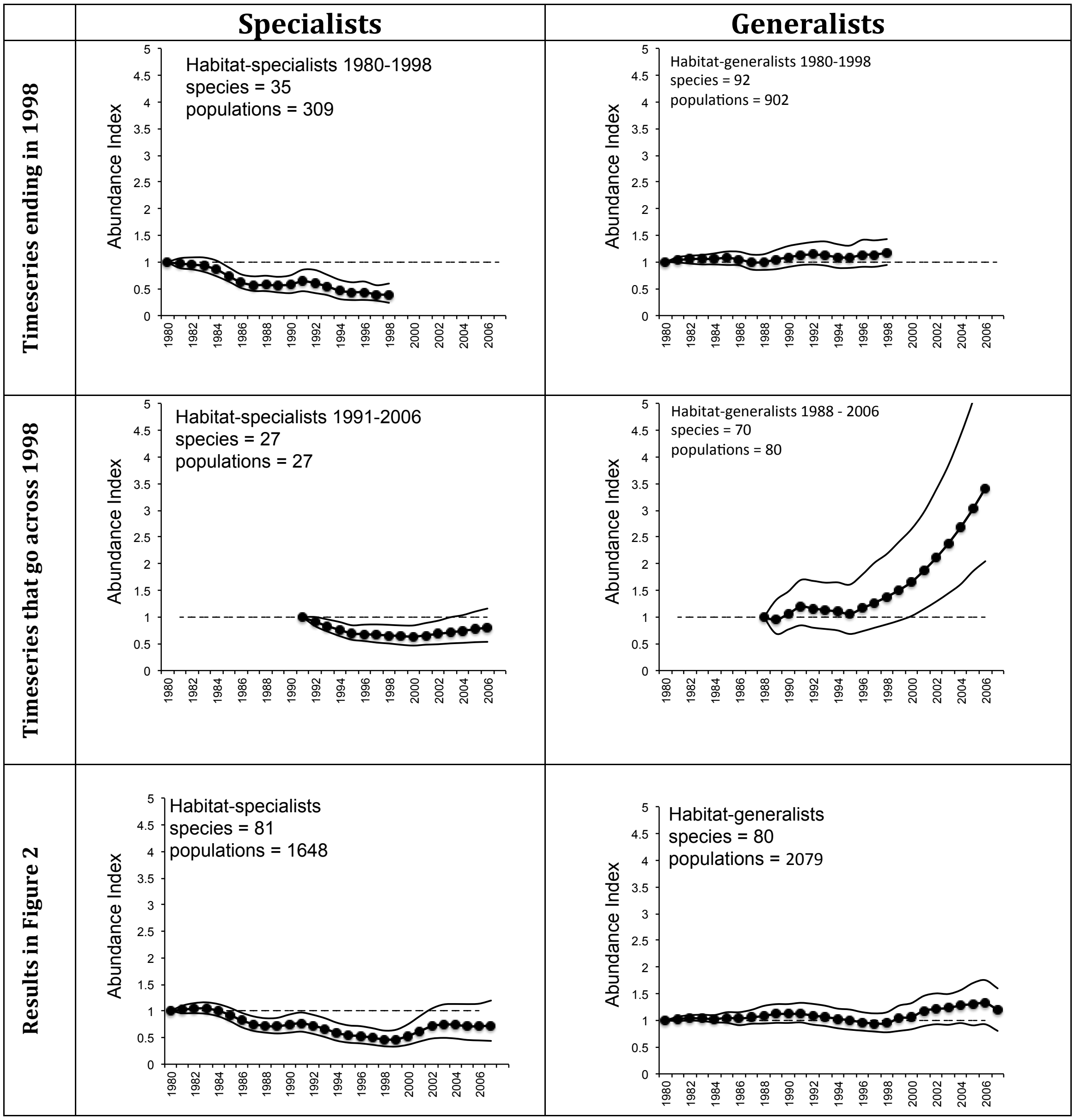


Figure S3. Temporal trends in the Abundance Index (+/- 95% CI, see Methods for calculations) of habitat-specialist (left column) and habitat-generalist (right column) Caribbean reef-fish species in long time-series that either stop in 1998 (top row) or span 1998 (middle row). The baseline year is indicated by a dashed line. Panels (A) and (B) from Figure 2 are shown to facilitate the visual interpretation.
