# Supplementary material for: Simplification of Caribbean Reef-Fish Assemblages over Decades of Coral Reef Degradation
Source: PLoS One. 2015 Apr 14;10(4):e0126004. doi: 10.1371/journal.pone.0126004 (PMC4397080; doi:10.1371/journal.pone.0126004)
Supplement: S4 Fig — A) Region-wide changes in mean coral cover and reef rugosity based on a meta-analysis of ecological studies across the Caribbean from 1977 to 2008 (Redrawn from Alvarez-Filip et al. 2011; Global Change Biol. 17:2470–2477). B) Temporal trends in the overall abundance of habitat-specialist fishes, relative to 1980 as depicted by the Abundance Index (Redrawn from Fig 2A). The grey dotted line indicates the year 2000, when at the regional scale, the abundance of habitat-specialist fishes started to recover but coral cover and reef rugosity continued to decline. The two panels derive from different data sources due to the lack of site overlap between the habitat (coral cover and reef rugosity) and fish datasets. (PDF) [file pone.0126004.s005.pdf]

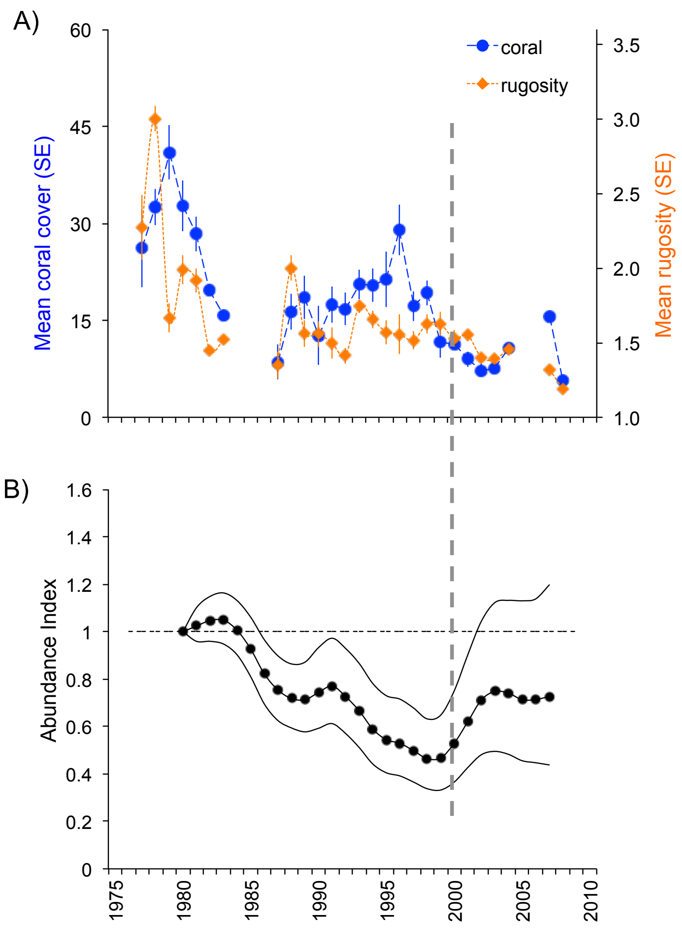

Figure S4. Long-term trajectories of change in coral cover, reef rugosity and habitat-specialist fishes on Caribbean reefs. A) Region-wide changes in mean coral cover and reef rugosity based on a meta-analysis of ecological studies across the Caribbean from 1977 to 2008 (Redrawn from Alvarez-Filip et al. 2011; *Global Change Biol.* 17:2470-2477). B) Temporal trends in the overall abundance of habitat-specialist fishes, relative to 1980 as depicted by the Abundance Index (Redrawn from Figure 2A). The grey dotted line indicates the year 2000, when at the regional scale, the abundance of habitat-specialist fishes started to recover but coral cover and reef rugosity continued to decline. The two panels derive from different data sources due to the lack of site overlap between the habitat (coral cover and reef rugosity) and fish datasets.
