# Supplementary material for: Simplification of Caribbean Reef-Fish Assemblages over Decades of Coral Reef Degradation
Source: PLoS One. 2015 Apr 14;10(4):e0126004. doi: 10.1371/journal.pone.0126004 (PMC4397080; doi:10.1371/journal.pone.0126004)
Supplement: S5 Fig — Baseline year is 1986 (dashed line at y = 1). (PDF) [file pone.0126004.s006.pdf]

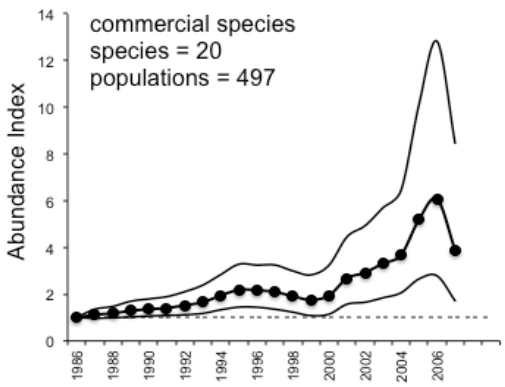

Figure S7. Temporal trends in aggregate abundance (Abundance Index, with 95% CI) of two major taxa of commercially important Caribbean reef fishes: Serranidae (groupers) and Lutjanidae (snappers). Baseline year is 1986 (dashed line at y = 1).
