# Supplementary material for: Simplification of Caribbean Reef-Fish Assemblages over Decades of Coral Reef Degradation
Source: PLoS One. 2015 Apr 14;10(4):e0126004. doi: 10.1371/journal.pone.0126004 (PMC4397080; doi:10.1371/journal.pone.0126004)
Supplement: S6 Fig — Between 1980 and 1988 all times series for inside MPAs are from only one study in Florida, USA. From 1991 to 2007, the number of studies contributing information for sites inside MPAs ranged between one and two, and represented only three other countries/territories (Saba, Costa Rica and Curaçao). Due to the scatter spatial and temporal distribution of the data, it was not possible to further explore the trends of change inside MPAs with the Abundance Index. (PDF) [file pone.0126004.s007.pdf]

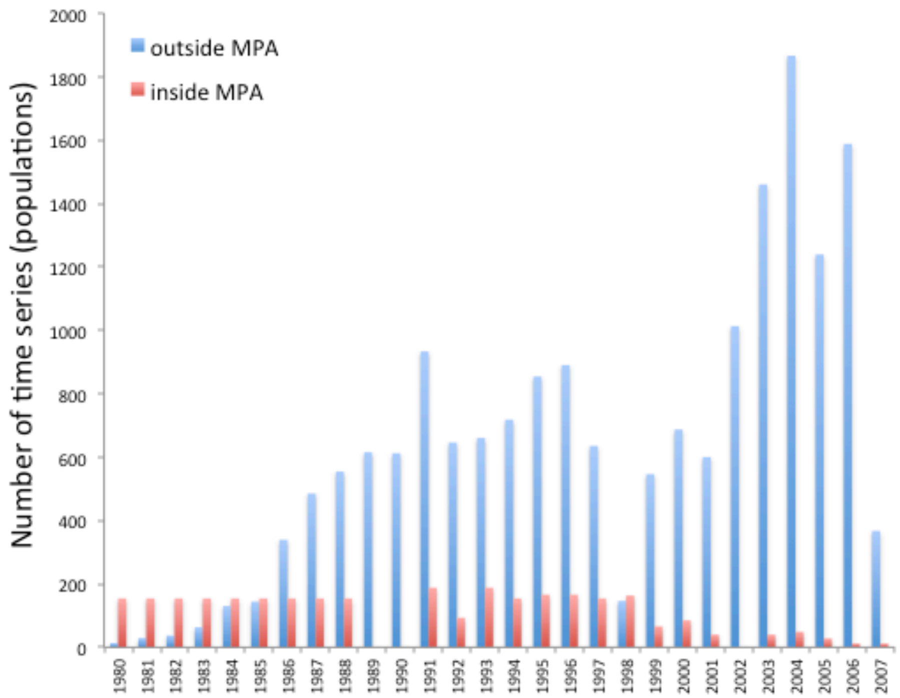

Figure S8. Number of populations (i.e., time series) collected from inside (red bars) and outside (blue bars) Marine Protected Areas in the Caribbean in each year of the study. Between 1980 and 1988 all times series for inside MPAs are from only one study in Florida, USA. From 1991 to 2007, the number of studies contributing information for sites inside MPAs ranged between one and two, and represented only three other countries/territories (Saba, Costa Rica and Curaçao). Due to the scatter spatial and temporal distribution of the data, it was not possible to further explore the trends of change inside MPAs with the Abundance Index.
