# Supplementary material for: Simplification of Caribbean Reef-Fish Assemblages over Decades of Coral Reef Degradation
Source: PLoS One. 2015 Apr 14;10(4):e0126004. doi: 10.1371/journal.pone.0126004 (PMC4397080; doi:10.1371/journal.pone.0126004)
Supplement: S1 Table — Specialists use only coral reef habitats, and generalists use coral reefs as well as one or more other coastal habitats (classification from Luiz et al. 2012). Fishing status is from Paddack et al. (2009). See Methods for description. (PDF) [file pone.0126004.s008.pdf]

Table S1. Species included in this study, their habitat categorisation according to the use of reef habitats. Specialists use only coral reef habitats, and generalists use coral reefs as well as one or more other coastal habitats (classification from Luiz et al. 2012). Fishing status is from Paddack et al. (2009). See Methods for description.

Family	Species	Habitat use	Fishing status
Acanthuridae	*Acanthurus bahianus*	Generalist	Fished
Acanthuridae	*Acanthurus chirurgus*	Generalist	Fished
Acanthuridae	*Acanthurus coeruleus*	Generalist	Fished
Apogonidae	*Apogon binotatus*	Specialist	Unfished
Apogonidae	*Apogon townsendi*	Specialist	Unfished
Aulostomidae	*Aulostomus maculatus*	Generalist	Unfished
Balistidae	*Balistes vetula*	Generalist	Fished
Balistidae	*Canthidermis sufflamen*	Generalist	Fished
Balistidae	*Melichthys niger*	Specialist	Fished
Balistidae	*Xanthichthys ringens*	Specialist	Fished
Batrachoididae	*Amphichthys cryptocentrus*	Generalist	Fished
Blenniidae	*Ophioblennius macclurei*	Specialist	Unfished
Bothidae	*Bothus lunatus*	Generalist	Fished
Carangidae	*Carangoides ruber*	Generalist	Fished
Carangidae	*Caranx bartholomaei*	Generalist	Fished
Carangidae	*Caranx crysos*	Generalist	Fished
Carangidae	*Caranx hippos*	Generalist	Fished
Carangidae	*Caranx latus*	Generalist	Fished
Carangidae	*Caranx lugubris*	Generalist	Fished
Chaenopsidae	*Acanthemblemaria aspera*	Specialist	Unfished
Chaenopsidae	*Acanthemblemaria spinosa*	Specialist	Unfished
Chaetodontidae	*Prognathodes aculeatus*	Specialist	Unfished
Chaetodontidae	*Chaetodon capistratus*	Generalist	Unfished
Chaetodontidae	*Chaetodon ocellatus*	Generalist	Unfished
Chaetodontidae	*Chaetodon sedentarius*	Specialist	Unfished
Chaetodontidae	*Chaetodon striatus*	Generalist	Unfished
Cirrhitidae	*Amblycirrhitus pinos*	Specialist	Unfished
Diodontidae	*Diodon holocanthus*	Generalist	Fished
Diodontidae	*Diodon hystrix*	Generalist	Fished
Gobiidae	*Coryphopterus dicrus*	Generalist	Unfished
Gobiidae	*Coryphopterus glaucofraenum*	Generalist	Unfished
Gobiidae	*Gnatholepis thompsoni*	Generalist	Unfished
Gobiidae	*Microgobius carri*	Generalist	Unfished
Gobiidae	*Coryphopterus lipernes*	Specialist	Unfished
Gobiidae	*Coryphopterus personatus*	Specialist	Unfished
Gobiidae	*Elacatinus evelynae*	Specialist	Unfished
Gobiidae	*Elacatinus genie*	Specialist	Unfished

Gobiidae	*Elacatinus illecebrosus*	Specialist	Unfished
Gobiidae	*Elacatinus oceanops*	Specialist	Unfished
Grammatidae	*Gramma loreto*	Specialist	Unfished
Haemulidae	*Anisotremus moricandi*	Specialist	Unfished
Haemulidae	*Anisotremus surinamensis*	Specialist	Fished
Haemulidae	*Anisotremus virginicus*	Specialist	Fished
Haemulidae	*Haemulon album*	Generalist	Fished
Haemulidae	*Haemulon aurolineatum*	Specialist	Fished
Haemulidae	*Haemulon carbonarium*	Specialist	Fished
Haemulidae	*Haemulon chrysargyreum*	Generalist	Fished
Haemulidae	*Haemulon flavolineatum*	Generalist	Fished
Haemulidae	*Haemulon macrostomum*	Specialist	Fished
Haemulidae	*Haemulon parra*	Generalist	Fished
Haemulidae	*Haemulon plumierii*	Generalist	Fished
Haemulidae	*Haemulon sciurus*	Generalist	Fished
Haemulidae	*Haemulon striatum*	Specialist	Fished
Haemulidae	*Haemulon vittatum*	Generalist	Unfished
Holocentridae	*Holocentrus adscensionis*	Specialist	Fished
Holocentridae	*Holocentrus rufus*	Specialist	Fished
Holocentridae	*Myripristis jacobus*	Specialist	Fished
Holocentridae	*Neoniphon marianus*	Specialist	Fished
Holocentridae	*Sargocentron coruscum*	Specialist	Fished
Holocentridae	*Sargocentron vexillarium*	Specialist	Fished
Kyphosidae	*Kyphosus incisor/sectatrix*	Specialist	Fished
Labridae	*Bodianus rufus*	Specialist	Fished
Labridae	*Clepticus parrae*	Specialist	Unfished
Labridae	*Halichoeres bivittatus*	Generalist	Unfished
Labridae	*Halichoeres garnoti*	Generalist	Unfished
Labridae	*Halichoeres maculipinna*	Specialist	Unfished
Labridae	*Halichoeres pictus*	Specialist	Unfished
Labridae	*Halichoeres poeyi*	Generalist	Unfished
Labridae	*Halichoeres radiatus*	Generalist	Fished
Labridae	*Lachnolaimus maximus*	Generalist	Fished
Labridae	*Thalassoma bifasciatum*	Specialist	Unfished
Labrisomidae	*Malacoctenus macropus*	Generalist	Unfished
Labrisomidae	*Malacoctenus triangulatus*	Specialist	Unfished
Lutjanidae	*Lutjanus analis*	Generalist	Fished
Lutjanidae	*Lutjanus apodus*	Generalist	Fished
Lutjanidae	*Lutjanus griseus*	Generalist	Fished
Lutjanidae	*Lutjanus jocu*	Generalist	Fished
Lutjanidae	*Lutjanus mahogoni*	Generalist	Fished
Lutjanidae	*Lutjanus synagris*	Generalist	Fished
Lutjanidae	*Ocyurus chrysurus*	Generalist	Fished

Malacanthidae	*Malacanthus plumieri*	Generalist	Fished
Monacanthidae	*Cantherhines macrocerus*	Specialist	Unfished
Monacanthidae	*Cantherhines pullus*	Specialist	Unfished
Monacanthidae	*Monacanthus tuckeri*	Generalist	Unfished
Monacanthidae	*Aluterus scriptus*	Specialist	Fished
Mullidae	*Mulloidichthys martinicus*	Generalist	Fished
Mullidae	*Pseudupeneus maculatus*	Generalist	Fished
Muraenidae	*Gymnothorax miliaris*	Specialist	Unfished
Muraenidae	*Gymnothorax moringa*	Generalist	Fished
Ostraciidae	*Lactophrys bicaudalis*	Generalist	Unfished
Ostraciidae	*Lactophrys triqueter*	Specialist	Fished
Ostraciidae	*Acanthostracion polygonius*	Specialist	Fished
Ostraciidae	*Acanthostracion quadricornis*	Generalist	Fished
Pempheridae	*Pempheris schomburgkii*	Specialist	Unfished
Pomacanthidae	*Centropyge argi*	Generalist	Unfished
Pomacanthidae	*Holacanthus bermudensis*	Specialist	Fished
Pomacanthidae	*Holacanthus ciliaris*	Specialist	Fished
Pomacanthidae	*Holacanthus tricolor*	Specialist	Unfished
Pomacanthidae	*Pomacanthus arcuatus*	Generalist	Fished
Pomacanthidae	*Pomacanthus paru*	Generalist	Fished
Pomacentridae	*Abudefduf saxatilis*	Specialist	Fished
Pomacentridae	*Abudefduf taurus*	Specialist	Unfished
Pomacentridae	*Chromis cyanea*	Specialist	Unfished
Pomacentridae	*Chromis insolata*	Specialist	Unfished
Pomacentridae	*Chromis multilineata*	Specialist	Unfished
Pomacentridae	*Chromis scotti*	Specialist	Unfished
Pomacentridae	*Microspathodon chrysurus*	Specialist	Unfished
Pomacentridae	*Stegastes diencaeus*	Specialist	Unfished
Pomacentridae	*Stegastes adustus*	Specialist	Unfished
Pomacentridae	*Stegastes leucostictus*	Generalist	Unfished
Pomacentridae	*Stegastes partitus*	Specialist	Unfished
Pomacentridae	*Stegastes planifrons*	Specialist	Unfished
Pomacentridae	*Stegastes variabilis*	Specialist	Unfished
Priacanthidae	*Heteropriacanthus cruentatus*	Specialist	Fished
Priacanthidae	*Priacanthus arenatus*	Specialist	Fished
Scaridae	*Scarus coelestinus*	Specialist	Fished
Scaridae	*Scarus coeruleus*	Generalist	Fished
Scaridae	*Scarus guacamaia*	Generalist	Fished
Scaridae	*Scarus iseri*	Generalist	Fished
Scaridae	*Scarus taeniopterus*	Specialist	Fished
Scaridae	*Scarus vetula*	Specialist	Fished
Scaridae	*Sparisoma atomarium*	Generalist	Unfished
Scaridae	*Sparisoma aurofrenatum*	Generalist	Fished

Scaridae	*Sparisoma chrysopterum*	Generalist	Fished
Scaridae	*Sparisoma radians*	Generalist	Unfished
Scaridae	*Sparisoma rubripinne*	Generalist	Fished
Scaridae	*Sparisoma viride*	Specialist	Fished
Sciaenidae	*Equetus punctatus*	Specialist	Fished
Sciaenidae	*Odontoscion dentex*	Generalist	Fished
Sciaenidae	*Pareques acuminatus*	Generalist	Fished
Serranidae	*Cephalopholis cruentata*	Generalist	Fished
Serranidae	*Cephalopholis fulva*	Specialist	Fished
Serranidae	*Epinephelus adscensionis*	Specialist	Fished
Serranidae	*Epinephelus guttatus*	Specialist	Fished
Serranidae	*Epinephelus morio*	Generalist	Fished
Serranidae	*Epinephelus striatus*	Generalist	Fished
Serranidae	*Hypoplectrus aberrans*	Specialist	Unfished
Serranidae	*Hypoplectrus chlorurus*	Specialist	Unfished
Serranidae	*Hypoplectrus gemma*	Specialist	Unfished
Serranidae	*Hypoplectrus gummigutta*	Specialist	Unfished
Serranidae	*Hypoplectrus guttavarius*	Specialist	Unfished
Serranidae	*Hypoplectrus indigo*	Specialist	Unfished
Serranidae	*Hypoplectrus nigricans*	Specialist	Unfished
Serranidae	*Hypoplectrus puella*	Generalist	Unfished
Serranidae	*Hypoplectrus unicolor*	Specialist	Unfished
Serranidae	*Liopropoma rubre*	Specialist	Unfished
Serranidae	*Mycteroperca bonaci*	Generalist	Fished
Serranidae	*Mycteroperca interstitialis*	Generalist	Fished
Serranidae	*Mycteroperca tigris*	Specialist	Fished
Serranidae	*Paranthias furcifer*	Specialist	Fished
Serranidae	*Rypticus saponaceus*	Generalist	Unfished
Serranidae	*Serranus baldwini*	Generalist	Unfished
Serranidae	*Serranus tabacarius*	Generalist	Unfished
Serranidae	*Serranus tigrinus*	Generalist	Unfished
Serranidae	*Serranus tortugarum*	Generalist	Unfished
Sparidae	*Calamus bajonado*	Generalist	Fished
Sparidae	*Calamus calamus*	Generalist	Fished
Sparidae	*Calamus penna*	Generalist	Fished
Sparidae	*Calamus pennatula*	Generalist	Fished
Synodontidae	*Synodus intermedius*	Generalist	Unfished
Tetraodontidae	*Canthigaster rostrata*	Specialist	Unfished
